# Tiotropium/Olodaterol treatment reduces cigarette smoke extract-induced cell death in BEAS-2B bronchial epithelial cells

**DOI:** 10.1186/s40360-020-00451-0

**Published:** 2020-10-31

**Authors:** Cheng-hsiung Chen, Yi-Rong Li, Sheng-Hao Lin, Hsiu-Hui Chang, Woei-Horng Chai, Po-Chiang Chan, Ching-Hsiung Lin

**Affiliations:** 1 Division of Chest Medicine, Department of Internal Medicine, Changhua Christian Hospital, 135 Nanhsiao Street, Changhua, 50006 Taiwan, Republic of China; 2 Changhua Christian Hospital, Thoracic Medicine Research center, Changhua, 50006 Taiwan, Republic of China; 3 Department of Recreation and Holistic Wellness, MingDao University, Changhua, Taiwan, Republic of China; 4 Institute of Genomics and Bioinformatics, National Chung Hsing University, Taichung, Taiwan, Republic of China

**Keywords:** CSE-induced cell death, Human bronchial epithelial cell, Tiotropium/olodaterol, Autophagy, JNK

## Abstract

**Background:**

Cigarette smoking is a critical risk factor for the destruction of lung parenchyma or the development of emphysema, which is characteristic of COPD. Disruption of epithelial layer integrity may contribute to lung injury following cigarette smoke extract (CSE) exposure. Tiotropium/olodaterol acts as a bronchodilator for COPD treatment; however, the effect of dual bronchodilators on epithelial cell injury and its underlying mechanism remain unclear. In this study, we evaluated the effect of tiotropium/olodaterol on CSE-mediated cell death and the underlying mechanisms.

**Methods:**

Cell viability was determined using the 3-(4,5-dimethylthiazol-2-yl)-2,5-diphenyltetrazolium bromide (MTT) assay. Apoptosis, necrosis, and autophagy were evaluated using flow cytometry. Autophagy-related protein, phosphorylated ERK, expression was determined using Western blotting.

**Results:**

Tiotropium/olodaterol significantly inhibited CSE-induced cell death, mitochondria dysfunction, and autophagy, which had no significant effect on apoptosis or necrosis in BEAS-2B human bronchial epithelial cells. Moreover, tiotropium/olodaterol attenuated CSE-induced upregulation of JNK.

**Conclusions:**

CSE induced cell death and caused consistent patterns of autophagy and JNK activation in BEAS-2B human bronchial epithelial cells. Tiotropium/olodaterol treatment protected bronchial epithelial cells from CSE-induced injury and inhibited activation of autophagy and upregulation of JNK phosphorylation. These results indicate that tiotropium/olodaterol may protect epithelial cells from the deleterious effects of CSE exposure, which is associated with the regulation of autophagy and JNK activation.

## Background

Cigarette smoke (CS) is a major risk factor for chronic obstructive pulmonary disease (COPD), which is characterized by emphysema and small airway obstruction, causing irreversible airflow limitation [1, 2]. Studies have reported that smokers have a faster FEV_1_ decline than nonsmokers. Moreover, the severity of COPD is correlated with the extent of cigarette smoking [3]. Cigarette smoking is known to induce cell death in lung structural cells, which contribute to the development of pulmonary emphysema in the lungs of smokers [4, 5]. However, the underlying mechanism of CS extract (CSE)-induced cell death remains unclear, despite evidence suggesting that cell death plays a crucial role in emphysema and COPD development.

In alveoli, epithelial cells form an essential barrier for the preservation of pulmonary function. As the first defense lining of the respiratory tract, the human bronchial epithelium is inevitably exposed to CS [6, 7]. Epithelial cells are vulnerable to insults from CS exposure through the induction of apoptosis and autophagy [8, 9]. Therefore, protecting bronchial epithelial cells against CS-induced alterations is paramount.

Studies have indicated that M3R and β2AR are involved in the mechanism of cell death. For instance, β2-Aabs levels appear to be associated with higher alveolar airspace destruction and aggravated smoking-related lung injuries [10]. Furthermore, M3 mAChR activation has been reported to inhibit cell proliferation [11]. These findings suggest that cell death can be prevented through the regulation of muscarinic receptors and β2AR. Tiotropium and olodaterol are bronchodilators that regulate M3R and β2AR, leading to the contraction and relaxation of the airway smooth muscle (ASM), respectively. Tiotropium/olodaterol is a long-acting muscarinic antagonist (LAMA) and long-acting β2-agonist (LABA) inhalation spray that is approved in several countries for the long-term maintenance treatment of COPD. As a dual bronchodilator, tiotropium/olodaterol has been reported to reduce ROS generation, which has been indicated as cell death inducers [12]. The role of dual bronchodilators on CSE-induced cell death in the human bronchial epithelium and the underlying mechanism remain unclear. We hypothesized that dual bronchodilators may contribute to the protection of epithelial cells against CSE-induced cell death. We investigated the effect of tiotropium/olodaterol on CSE-mediate epithelial injury and the possible mechanisms of action.

## Methods

### Chemicals and reagents

Tiotropium and olodaterol were purchased from Tocris (Ellisville, MO, USA). Acridine orange (N,N,N′,N′-tetramethylacridine-3,6-diamine), 5,5′,6,6′-tetrachloro-1,10,3,3′-tetraethylbenzimidazolylcarbocyanine iodide (JC-1; Sigma-Aldrich, St. Louis, MO, USA), and 3-(4,5-dimethylthiazol-2-yl)-2,5-diphenyltetrazolium bromide (MTT) were purchased from (St. Louis, MO, USA). All the chemicals were dissolved in DMSO and stored at − 20 °C. MTT, JC-1, and acridine orange were dissolved in water and stored at 4 t.

### Antibodies

Anti-LC-3 and Anti-Beclin 1 were purchased from Novus Biological (Littleton, CO, USA). Anti-phospho-ERK and anti-phospho-JNK were purchased from Santa Cruz (Santa Cruz, CA, USA). GAPDH was purchased from Cell signaling (Danvers, MA, USA). Goat anti-rabbit IgG and goat anti-mouse IgG were purchased from Jackson Laboratory (Bar Harbor, ME, USA).

### Cell culture

BEAS-2B (human bronchial epithelial cell line;. ATCC, CRL-9609) were cultured in RPMI 1640 medium containing 10% fetal bovine serum (Gibco, Gland Island, NY, USA) and 1% antibiotic antimycotic. The cells were maintained in an incubator at 37 °C with a humidified atmosphere with 5% CO_2_. The cells were grown to 90% confluence and passaged by trypsin/EDTA.

### Cigarette smoke extraction

CSE was prepared as previously described with minor modifications [13, 14]. In brief, commercial cigarettes (LONG LIFE, Taiwan), containing 1.2 mg of nicotine and 12 mg of tar, were used to obtain CSE. The smoke of five cigarettes was bubbled through 10 mL of PBS. The solution was considered to have 100% strength CSE. The peristaltic pump was equilibrated at a rate of one cigarette per 5 min. The CSE suspension was then filtered through a 0.22-μm pore filter to remove bacteria and particles.

### Cell viability assay

BEAS-2B cells were seeded in 24-well plates at a density of 2 × 10^4^ cells/mL and were pretreated with or without various concentrations of tiotropium/olodaterol for 4 h. The cells were then cultured with 0–10% CSE for 24 h. After 24-h incubation, 200 μL of 1 mg/mL MTT in RPMI 1640 was added to each well at 4 h before the end of each incubation. The MTT solution was then removed. The adherent cells were lysed with 600 μL of DMSO, and the optical density was obtained at 570 nm using a microplate reader (PerkinElmer).

### Detection of the mitochondrial membrane potential

BEAS-2B cells were seeded in 6-well plates at a density of 2 × 10^5^ cells/mL and pretreated with or without various concentrations of tiotropium/olodaterol for 4 h. The cells were then cultured with 5% CSE for 24 h. After incubation, the cells were removed from the plate with trypsin-EDTA (GIBCO-BRL), and 10 μg/mL JC-1 (Sigma-Aldrich, St. Louis, MO, USA) and serum-free medium were mixed at a ratio of 1:500, and 1 mL of the mixture was added to each sample, followed by incubation at 37 °C for 15 min. The samples were detected and quantified using an Aria III flow cytometer (BD Biosciences).

### Annexin V assay

BEAS-2B cells were cultured at a density of 2 × 10^5^ cells/mL in a 6-well plate (Corning Glass Works, Corning, NY, USA). The next day, the cells were then pretreated with 10 μM tiotropium/olodaterol combined 12.5 or 25 μM tiotropium for 4 h followed by 5% CSE exposure for 24 h. After the incubation period, the cells were collected by trypsin-EDTA treatment and centrifugation. Apoptotic cell quantification was performed using an annexin V-FITC apoptosis detection kit (BD Biosciences, Franklin Lakes, NJ, USA). The cells were stained following the annexin V-FITC/PI staining protocol for 15 min at RT. The fluorescence of annexin V/PI was then detected with the Aria III flow cytometer (BD Biosciences).

### Acridine orange assay

Autophagy is characterized by the formation of acidic vesicular organelles (AVOs). We used acridine orange to characterize the acidic cellular compartment that emits bright red fluorescence in acidic vesicles and green fluorescence in the cytoplasm and nucleus. BEAS-2B cells were seeded in 6-well plates at a density of 2 × 10^5^ cells/mL and pretreated with and without various concentrations of tiotropium/olodaterol for 4 h. The cells were then cultured with 5% CSE for 6 and 24 h. After incubation, the cells were removed from the plate with trypsin-EDTA (GIBCO-BRL). Acridine orange was added at a final concentration of 1 mg/mL, and the plates were then incubated at 37 °C for 15 min. The AVOs in BEAS-2B cells were detected and quantified using the Aria III flow cytometer (BD Biosciences). The AVOs displayed bright red fluorescence (650 nm, FL-3 channel), whereas the cytoplasm and nucleolus displayed green fluorescence (500–550 nm, FL-1 channel) [10]. The intensity of red fluorescence was proportional to the number of AVOs in autophagic cells.

### Western blotting

The cells were lysed in RIPA buffer with the 10% proteasome inhibitor. The cell extracts were cleared at 12000 rpm in a microcentrifuge at 4 °C for 20 min. Proteins were separated by 10 and 15% SDS-PAGE and transferred onto a PVDF membrane. The membrane was blocked in 5% nonfat dry milk in Tris-buffered saline with Tween 20 (TBST) buffer. Immunostaining was then performed using anti-LC-3, anti-Beclin 1, anti-phospho-ERK, or anti-phospho-JNK, followed by incubation with HRP-conjugated anti-rabbit or anti-mouse IgG secondary antibodies. ECL reagent (GE Healthcare Life Sciences, Chalfont, UK) was used for protein detection.

### Statistical analysis

All values are expressed as mean ± SD. For comparison between two groups, we used unpaired two-tailed *t* tests (Student’s *t* tests). *P* values of < 0.05 were considered statistically significant. Data were analyzed using GraphPad Prism Version 6.0 (San Diego, CA, USA).

## Results

### CSE induces death in BEAS-2B bronchial epithelial cells

**Fig. 1:**
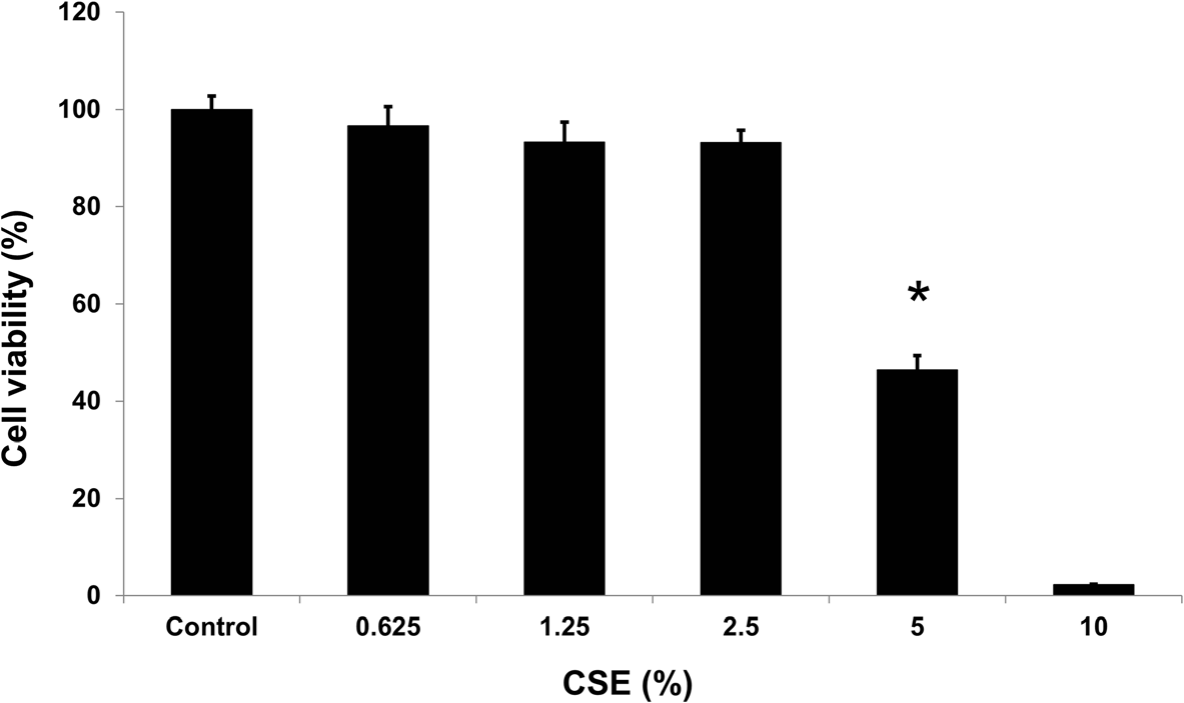
Effects of cigarette smoke extraction (CSE) on the viability of BEAS-2B cells. Cell viability of BEAS-2B cells after treatment with various concentrations of CSE for 24 h. Cell viability was determined using the MTT assay. The absorbance of the reaction solution at 570 nm was recorded. Data are presented as means ± SD from triplicate samples for each treatment. *P < 0.05 versus DMSO-treated control

To evaluate the effect of CSE on BEAS-2B bronchial epithelial cells, BEAS-2B cells were exposed to various dosages of CSE. As illustrated in Fig. 1, CSE treatment significantly reduced cell viability after 24-h treatment with 5% CSE and 10% CSE. The IC_50_ was approximately 5% CSE. Doses lower than 2.5% CSE exhibited slight toxicity compared with doses above 2.5%. These results indicate that CSE treatment considerably increased bronchial cell injury at a dose greater than 5% CSE.

### Tiotropium/olodaterol treatment reduces CSE-induced cell death in BEAS-2B bronchial epithelial cells

**Fig. 2:**
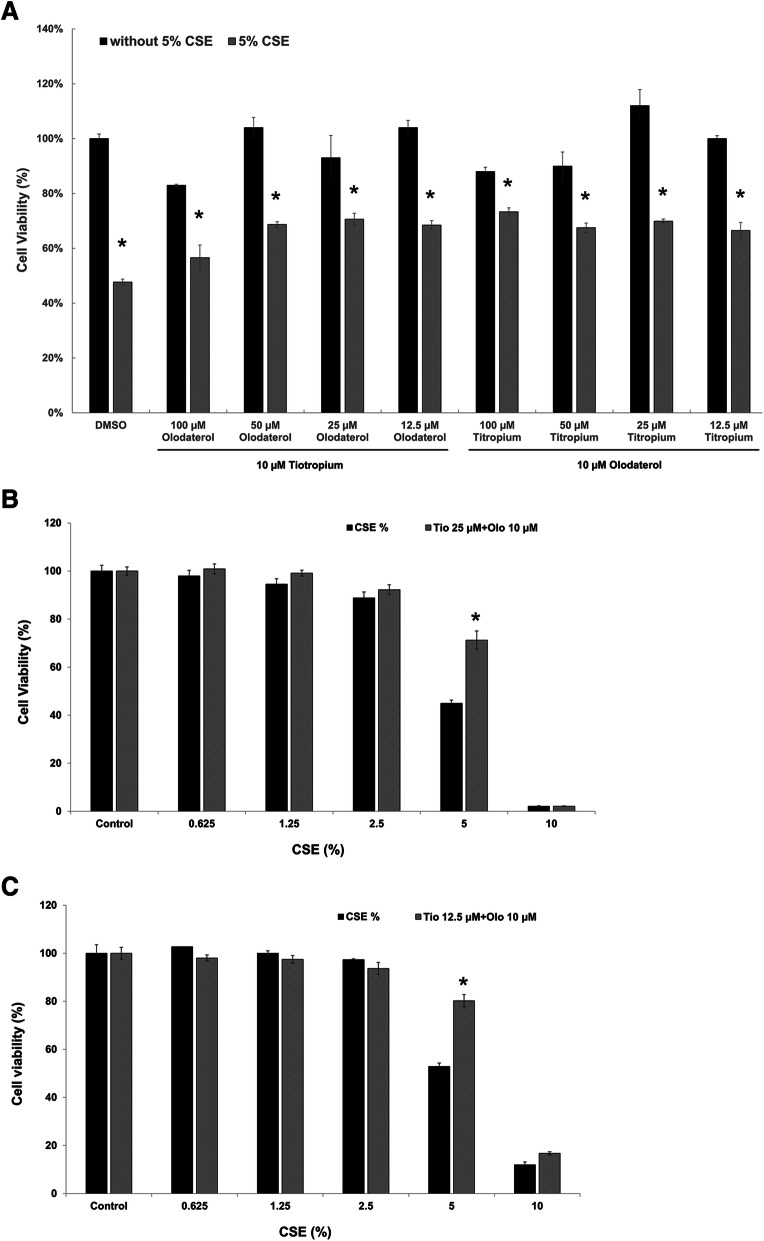
Effects of tiotropium/olodaterol on CSE-induced cell death in BEAS-2B cells. **a** Cell viability of BEAS-2B cells after pretreatment with 25 μM tiotropium + 10 μM Olodaterol for 4 h, followed by CSE treatment for 24 h. **b** Cell viability of BEAS-2B cells after pretreatment with 25 μM tiotropium + 10 μM Olodaterol for 4 h, followed by CSE treatment for 24 h. Cell viability was determined using the MTT assay. The absorbance of the reaction solution at 570 nm was recorded. Data are presented as means ± SD from triplicate samples for each treatment. *P < 0.05 versus 5% CSE-treated group. Tio: tiotropium; Olo: Olodaterol

To evaluate the effect of tiotropium/olodaterol on CSE-induced epithelial cell death, we pretreated the cells with various combinations of tiotropium/olodaterol for 4 h, followed by 5% CSE treatment for 24 h, and cell viability were determined using the MTT assay. As illustrated in Fig. 2a, after pretreatment with bronchodilators at various combination dosages, combined olodaterol (10 μM) and tiotropium (12.5 or 25 μM) treatment significantly increased cell viability after 5% CSE exposure whish is minimal dosage and have no harmfulness in condition of without 5% CSE exposure. Therefore, the combination of 10 μM olodaterol and 12.5 or 25 μM tiotropium was selected as the ideal treatment for further experiments in this study. As illustrated in Fig. 2b and c, pretreatment with tiotropium/olodaterol (10 μM olodaterol combined with 12.5 or 25 μM tiotropium) enhanced cell survival after 5% CSE exposure compared with 5% CSE exposure alone. These results indicate that pretreatment with BD has a protective effect against cell injury caused by CSE exposure.

### Tiotropium/olodaterol treatment has no significant effect on apoptosis and necrosis in BEAS-2B bronchial epithelial cells after CSE exposure

**Fig. 3:**
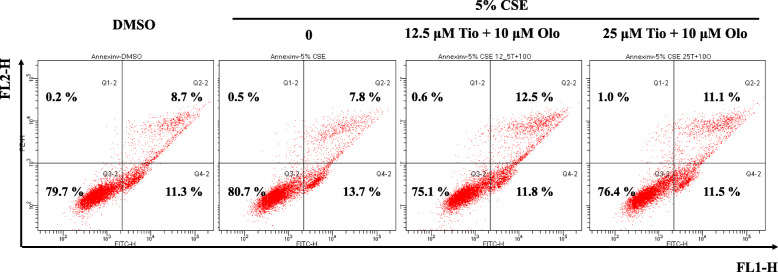
The effect of tiotropium/olodaterol on apoptosis and necrosis in BEAS-2B bronchial epithelial cells after CSE exposure. BEAS-2B cells were pretreated with tiotropium/olodaterol for 4 h, followed by treatment with or without 5% CSE for 24 h. The cells were then stained with annexin V-FITC and propidium iodide (PI) and analyzed using flow cytometry. Tio: tiotropium; Olo: Olodaterol

To clarify the effect of tiotropium/olodaterol on apoptosis and necrosis following CSE exposure, BEAS-2B cells were pretreated with tiotropium/olodaterol and subjected to flow cytometric analysis after annexin V-FITC and PI staining. As illustrated in Fig. 3, flow cytometric analysis demonstrated that the percentages of early apoptotic (annexin V+/PI−, lower right quadrant) and late apoptotic (annexin V+/PI+, upper right quadrant) BEAS-2B cells increased with exposure to 5% CSE for 24 h, without necrotic cell death (annexin V−/PI+, upper left quadrant). Pretreatment with tiotropium/olodaterol (10 μM olodaterol combined with 12.5 or 25 μM tiotropium) had no significant effect on the percentage of apoptotic and necrotic cell death compared with 5% CSE exposure alone. These data suggest that the inhibition of apoptosis or necrosis may not be involved in the protective effect of tiotropium/olodaterol against CSE-induced cell death.

**Fig. 4:**
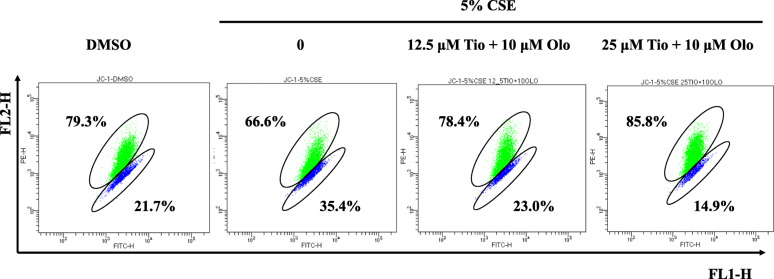
Effect of Tiotropium/olodaterol on CSE-mediated MMP change in BEAS-2B cells. BEAS-2B cells were pretreated with tiotropium/olodaterol for 4 h, followed by treatment with or without 5% CSE for 24 h. The cells were then stained with JC-1 and analyzed using flow cytometry. FL1-H fluorescence represents those with a depolarized mitochondrial membrane, and FL2-H fluorescence represents cells with normal mitochondria membrane potential. Tio: tiotropium; Olo: Olodaterol

### Tiotropium/olodaterol treatment reverses CSE-induced mitochondria membrane potential disruption in BEAS-2B bronchial epithelial cells

Changes in mitochondrial membrane potential (ΔΨm) are integral to the cell life–death transition. To evaluate the effect of tiotropium/olodaterol on mitochondria membrane potential disruption upon CSE exposure, treated cells were stained with the fluorescent cationic dye JC-1. Loss of ΔΨm is an indicator of mitochondrial damage during cell death. After the cells were treated with 5% CSE for 24 h, FL1 fluorescence was detected in 34.5% of BEAS-2B cells, which is higher than the percentage in the control group, suggesting that 5% CSE causes a reduction in ΔΨm. A decrease in the FL1 fluorescence intensity was observed in the group pretreated with tiotropium/olodaterol (10 μM olodaterol combined with 12.5 or 25 μM tiotropium) (Fig. 4). These findings indicate that tiotropium/olodaterol treatment delays CSE-induced mitochondrial dysfunction.

### Tiotropium/olodaterol treatment inhibits CSE-induced autophagy in BEAS-2B bronchial epithelial cells

**Fig. 5:**
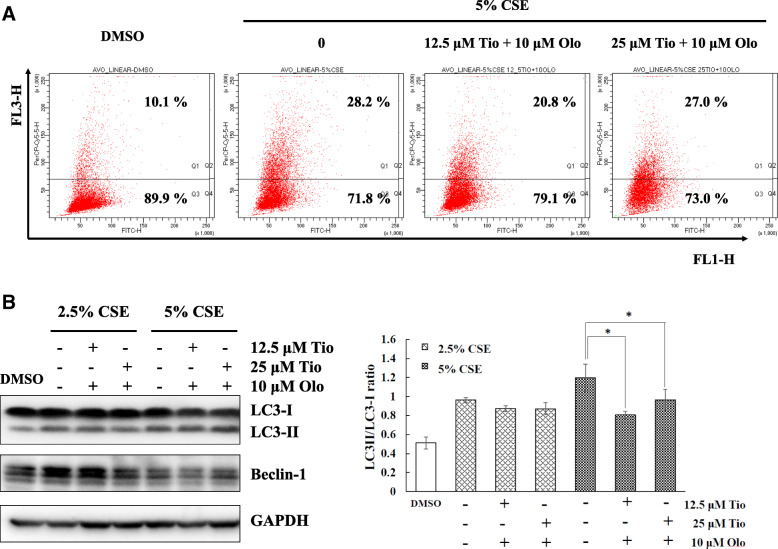
Effect of Tiotropium/olodaterol on CSE-mediated autophagy in BEAS-2B cells in long term treatment. **a** BEAS-2B cells were pretreated with tiotropium/olodaterol for 4 h, followed by treatment with or without 2.5 and 5% CSE for 24 h. AVO staining and flow cytometry analysis were then performed. AO accumulates and aggregates are characterized by yellow-orange fluorescence, and normal cells with AO are characterized by green fluorescence. **b** Western blot analysis of the expression of LC3 and Beclin 1 in BEAS-2B cells. *P < 0.05 versus 5% CSE-treated group. Tio: tiotropium; Olo: Olodaterol

**Fig. 6:**
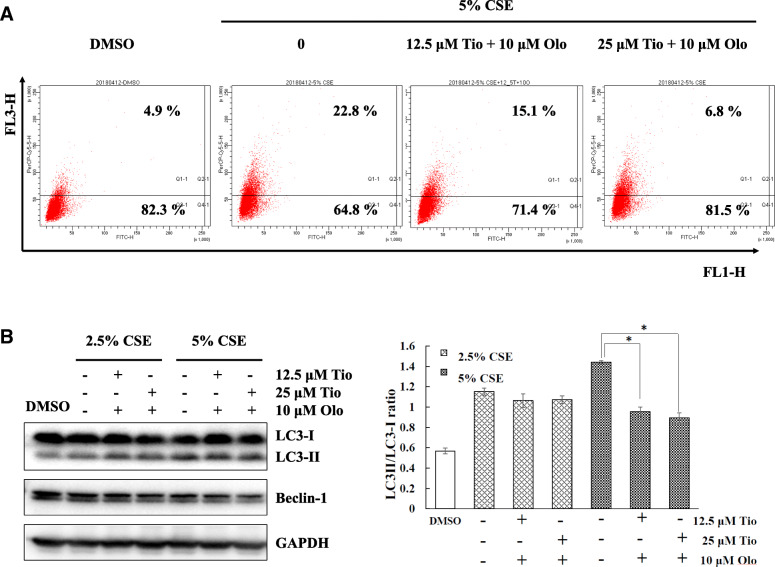
Effect of Tiotropium/olodaterol on CSE-mediated autophagy in BEAS-2B cells in short term treatment. **a** BEAS-2B cells were pretreated with tiotropium/olodaterol for 4 h, followed by treatment with or without 2.5 and 5% CSE for 6 h. AVO staining and flow cytometry analysis were then performed. AO accumulates and aggregates are characterized by yellow-orange fluorescence, and normal cells with AO are characterized by green fluorescence. **b** Western blot analysis of the expression of LC3 and Beclin 1 in BEAS-2B cells. *P < 0.05 versus 5% CSE-treated group. Tio: tiotropium; Olo: Olodaterol

For autophagolysosomes, the protonated form of AO accumulates and aggregates, which is characterized by yellow-orange fluorescence (FL3). The staining of normal cells with AO, a weak base, is characterized by green fluorescence (FL1). To explore the effect of tiotropium/olodaterol on autophagy following CSE exposure, BEAS-2B cells were pretreated with tiotropium/olodaterol and subjected to flow cytometric analysis after AVO staining. As illustrated in Fig. 5a, 5% CSE exposure activates the autophagic process, as demonstrated by the marked increase in the appearance of yellow-orange (FL-3) fluorescence in BEAS-2B cells and the increased conversion of LC3-I to LC3-II in BEAS-2B cells in Western blot analysis at 24 h after CSE exposure **(**Fig. 5b). However, pretreatment with tiotropium/olodaterol (10 μM olodaterol combined with 12.5 μM tiotropium) significantly attenuated the induction of autophagy without changing the basal level of autophagy in 24-h CSE–treated cells compared with 5% CSE exposure alone. Notably, in short-term (6 h) exposed CSE cells, pretreatment with a combination of 10 μM olodaterol and 12.5 or 25 μM tiotropium significantly attenuated CSE-induced autophagy compared with 5% CSE exposure alone **(**Fig. 6a and b). These findings suggest that tiotropium/olodaterol has an inhibitory effect on CSE-induced autophagy, especially after short-term exposure.

### Tiotropium/olodaterol treatment induces ERK and JNK activation in BEAS-2B bronchial epithelial cells

**Fig. 7:**
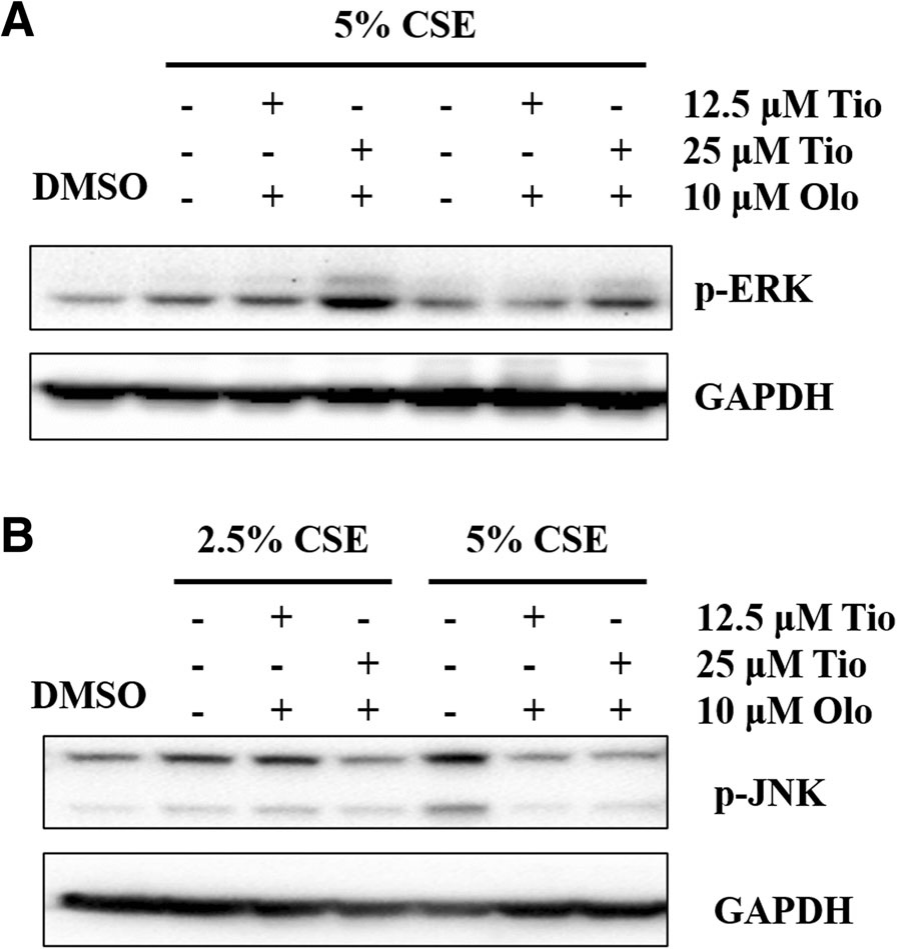
Effects of Tiotropium/olodaterol on ERK and JNK activation. **a** BEAS-2B cells were pretreated with Tiotropium/olodaterol for 4 h, followed by treatment with or without 2.5 and 5% CSE for 3 h. Western blot analysis of the expression of phosphor ERK in BEAS-2B was performed. **b** BEAS-2B cells were pretreated with Tiotropium/olodaterol for 4 h, followed by treatment with or without 5% CSE for 6 h. Western blot analysis of the expression of phosphor JNK in BEAS-2B. Tio: tiotropium; Olo: Olodaterol

ERK activation and JNK activation are involved in cell survival and cell death and autophagy [15, 16]. The phosphorylation levels of ERK and JNK were evaluated by Western blotting to further understand whether tiotropium/olodaterol can regulate ERK and JNK activation in CSE-treated BEAS-2B cells. As illustrated in Fig. 7a and b, JNK phosphorylation levels increased in 5% CSE-treated cells, whereas pretreatment with tiotropium/olodaterol (10 μM olodaterol combined with 12.5 μM tiotropium) effectively reduced the CSE-induced upregulation of p-JNK. However, tiotropium/olodaterol had no significant effect on ERK phosphorylation levels after 5% CSE exposure compared with 5% CSE exposure alone. These findings suggest that inhibition of JNK activation by tiotropium/olodaterol may contribute to the alteration of CSE-induced cell death and autophagy.

## Discussion

Cigarette smoking is the major cause of irreversible lung diseases, including COPD and emphysema. Increasing evidence has indicated that cigarette smoking–induced lung injury is related to epithelial cell disruption [6, 8, 9, 17]. A combination of tiotropium and olodaterol is used as long-term maintenance treatment for COPD, including chronic bronchitis or emphysema [18]. Tiotropium/olodaterol acts as a bronchodilator by preventing ASM contraction and inducing muscle relaxation through the regulation of muscarinic receptors and β2 adrenergic receptors, respectively [19]. Few studies have indicated that bronchodilators have a protective effect on CSE-induced lung epithelial cell injury because they reduce inflammatory responses [20, 21]. However, the effect of dual bronchodilators on CSE-induced cell injury in epithelial cells remains unclear. In the present study, we demonstrated that CSE exposure significantly reduced BEAS-2B bronchial epithelial cell survival, reduced mitochondria dysfunction, and markedly induced autophagy, but not apoptosis. To our knowledge, this is the first study to demonstrate that tiotropium/olodaterol protects BEAS-2B human bronchial epithelial cells against CSE-induced cell death.

Oxidative stress, inflammation, apoptosis, imbalance of protease–antiprotease, and autophagy are regarded as the principal contributors to the development of COPD [21]. Moreover, several studies have demonstrated that the inhibition of autophagy controls lung cell death and emphysema development caused by CS exposure in mice [4, 5]. This finding suggests that autophagy may be a critical target for regulating cigarette smoke–induced lung injury. However, few studies have reported the action of bronchodilator in modulating autophagy in lung epithelial cells. A study demonstrated that activation of β-arrestin2, downstream of β2 adrenergic receptors, contributes to the inhibition of autophagy, which engenders a decrease in inflammation in BEAS-2B cells [22]. Moreover, the muscarinic receptor antagonist atropine reduced ACh-induced autophagy [15]. These findings indicated that autophagy may be a crucial factor in the tiotropium/olodaterol modulation of epithelial cell death. Our findings demonstrated that tiotropium/olodaterol treatment reduced LC-3I conversion to LC-3II and AVO expression following CSE exposure, which displayed consistent patterns of cell survival. Moreover, tiotropium/olodaterol treatment ameliorated CSE-induced autophagy in both short-term (6 h) and long-term (24 h) CSE exposure. These findings suggest that the inhibition of autophagy by tiotropium/olodaterol treatment may act as an adaptive response against cell death, which contributes to the protection of epithelial cells in response to CSE exposure.

A study demonstrated that CS affects the mitochondrial adaptive stress response in alveolar epithelial cells, which may contribute to the pathogenesis of COPD [22]. Moreover, prolonged mitochondrial damage can induce excessive levels of mitophagy, thereby enhancing cell death and tissue injury [23]. We observed that CSE exposure induced slight early apoptosis and a significant increase in autophagy activation, which was consistent with the model of the loss of mitochondria membrane potential. Furthermore, tiotropium/olodaterol treatment did not affect CSE-induced apoptosis but did reduce autophagy activation. These findings suggested that tiotropium/olodaterol–attenuated CSE-induced mitochondria dysfunction may contribute to regulating autophagy instead of apoptosis, causing cell death.

ERK and JNK signaling pathways are involved in cell survival and cell death by regulating autophagy [15, 16]. However, the interactions between autophagy and activation of ERK and JNK are complex. ATG5, an autophagy-related protein, serves as a cellular scaffold to induce ERK phosphorylation [24]. Inhibition of JNK expression reduces Beclin 1 and represses autophagy [25]. However, sustained ERK activation may suppress the autophagic flux, and MAPK/JNK activation prevents the induction of autophagy by activating mTOR [26]. We determined that tiotropium/olodaterol significantly suppresses the activation of JNK, but not ERK, after CSE exposure. A study demonstrated that CSE-induced autophagy activation in bronchial epithelia acts in synergism with the ERK pathway and contributes to airway inflammation [27]. Another study suggested that JNK activation is also involved in CSE-induced autophagy in BEAS-2B human bronchial epithelial cells, which is in accordance with our findings [28]. Moreover, JNK activation promotes autophagy by releasing Beclin 1 from the BCL2–Beclin 1 complex [29, 30]. Our results demonstrated that tiotropium/olodaterol treatment reduced Beclin 1 expression (Figs. 5b and 6b) and reduced JNK phosphorylation (Fig. 7b) following CSE exposure. These results suggest that the inhibition of the JNK signaling pathway by tiotropium/olodaterol may contribute to preventing CSE-induced cell death and inhibiting autophagy in BEAS-2B bronchial epithelial cells.

The lung epithelium plays a crucial role in the lung’s response to CS, which is an active participant in emphysema and COPD pathogenesis [31]. The Global Initiative for COPD (GOLD) recommendations are that bronchodilators are the mainstay of pharmacotherapy for COPD. The use of dual bronchodilator with a LAMA and a LABA is considered when symptoms have not improved with monotherapy. The tiotropium/olodaterol fixed-dose combination is indicated in several countries for once-daily maintenance treatment of airflow obstruction in patients with COPD, including emphysema [32]. However, few studies have investigated the mechanism of the protective effect of tiotropium/olodaterol against CSE-induced cell death. M3R and β2AR are involved in regulating the mechanism of cell death [10, 11]. Furthermore, CSE promotes apoptosis of CD8+ T cells, which can be completely abrogated by muscarinic antagonists [33]. These findings suggest that muscarinic antagonists and β2-agonists protect against CSE-induced death. In the present study, we provide in vitro evidence to clarify the protective effects of tiotropium/olodaterol against CSE-induced epithelial cell injury and the possible mechanism, including activation autophagy and reduced mitochondria dysfunction. The effect of tiotropium/olodaterol on diseases in animal models requires further research.

In the current study, we demonstrated that tiotropium/olodaterol treatment prevented CSE-induced cell death, autophagy activation, and JNK activation in BEAS-2B human bronchial epithelial cells. Therefore, tiotropium/olodaterol could be used to protect epithelial cells from the deleterious effect of CSE exposure by regulating autophagy and JNK activation.

## Data Availability

The analyzed datasets generated during the study are available from the corresponding author upon reasonable request.

## Abbreviations

ATG5: Autophagy-related (ATG) proteins 5
ASM: Airway smooth muscle
β2AR: β2-adrenergic receptor
β2-Aabs: Agonistic β2-adrenergic receptor autoantibodies
COPD: Chronic obstructive pulmonary disease
CSE: Cigarette smoke extract
ERK: Extracellular signal-regulated kinase
FEV_1_: Forced expiratory volume in one second
JNK: c-Jun N-terminal kinase
LAMA: Long-acting muscarinic antagonist
LABA: Long-acting β2-agonist
M3R: Muscarinic acetylcholine receptor M3
MAPK: Mitogen-activated protein kinase
Olo: Olodaterol
ROS: Reactive oxygen species
Tio: Tiotropium
